# Proceedings of the National Medical Association

**Published:** 1870-05

**Authors:** 


### Editorial Department.

### Proceedings of the National Medical Association.

Our medical legislators having held their annual session, it is natural to sum up the amount of their decisions. It will not be supposed that in this calculation we consider at all the value of the various papers upon medical and surgical subjects, which were presented to the various "sections," and which will appear in the volume of transactions, and may be better estimated at another time. However, it cannot be supposed but that they are mostly valuable original contributions to medical and surgical science. Whatever may be said about the labors of the legislative "section," the other divisions are not included. There can be no division of sentiment as to the quality, amount, and valuation, of the contributions made to medicine and surgery through the medium of the American Medical Association. Again, the social "section" of the association, is entirely unexceptionable in its operations. To bring the members of the profession in closer sympathy with each other, to extend personal acquaintance, and to promote friendly intercourse, is the main object of this department. In its workings, it is one of the chief attractions; it is pleasant to meet face to face the *savans* of the profession, those who constitute much of its strength, and honor, and glory. To observe the anxious, restless, untiring, never ceasing enthusiasm of the progressive, ambitious aspirants for distinction, is itself a healthful stimulant, since men sometimes profit by example. Yes, the social "section" has its pleasures and advantages, and its labors should be commended and continued.

We proposed to say a word about the legislative proceedings of the association; that division, which for harmony we will denominate the legislative "section." ☞ These new "sections," we hope, will all be hereafter recognized by the association. ☜ This legislative "section" consists of a committee of the whole, and has truly a *great work* to perform. In attempting a brief description of its operations at the recent meeting in Washington, we shall be obliged to give only brief outlines taken from indistinct recollections, after observing the exhibition for a few days only. This committee of the whole was organized for legislative labor in the forenoon only, the regular officers of the association presiding. In the center of the stage stood the president with a small mallet in his hand, supported on either side by vice-presidents, secretaries, and reporters, while the rear guard was formed of the venerable men of the profession, whom we all love and honor. The president shortly announced that the meeting was open for business, and that first in the order would be, *Report* from Business Committee. Pending the reading of this report, prayer was offered, during which, it being very brief, little else was attempted, except some caucussing in the upper part of the room and about the door. Prayer being said, Mr. Chairman, of Business Committee, read the report, the first two or three sentences of which were listened to with marked attention. After its conclusion, provided it was concluded, a minority report was offered, and a few words of explanation attempted. Meanwhile the president commenced to pound feebly with his mallet and say, "order!" as near as I could understand; and about three hundred men followed, shouting, "out of order!" or something of that sort, while some very loud spoken individuals were constantly screaming: "Mr. President," "Mr. President," "Mr. President." Becoming exhausted, there was a period of collapse, which lasted about three-quarters of a second, when a very gentlemanly looking individual walked upon the stage, and commenced a speech, when all the entire audience attempted a speech—each upon his own account—and at a pitch of voice perfectly indiscribable; whereupon there was soon a division of the "section," some of the members continuing their speeches, and others shouting "Can't hear," while the remainder were either hissing, or clapping their hands, and hallowing: "Go on, that's right, go ahead." Says I to my neighbor, "What is this?" Looking astonished, he says: "Why, this is the meeting of the American Medical Association in open session." This was spoken in one ear, my nearest neighbor on the other side screaming in the other—"Louder, louder, louder!" At this juncture it occurred to me that as I was editor of a medical journal, I would take notes of the proceedings for publication, which I proceeded to do with great care, and should have been able to have presented them to my readers, as taken on the spot, had not the association voted that "no publications, or papers of any sort, be taken into or carried out of the hall;" whereupon, I threw my notes in with the certificates of members, thinking I would avoid all personal difficulties, even at the sacrifice of the wishes and interests of the readers of my favorite journal. The president now announced that next in the order of business was the reading of the President's Address, when nearly every man left the hall. These were the proceedings of the legislative "section," the first day of the session. The next morning being interested to know what would be the order of exercises, I went early to the hall. The first vote taken was to "adjourn until a suitable hall could be provided, as not one word could be heard in *that* hall." While I was taking lively satisfaction at the prospect of being able to hear again, not having been able to understand a word of common conversation since my neighbor had screamed so like a hyena in my ear the day before, the vote was reconsidered. One man, with a very loud voice, now said that "If the president would take his stand, or move his platform into the center of the hall, his mallet might be more distinctly felt." Fifty or sixty men now started to their feet, and simultaneously made suggestions, or motions, or offered resolutions about the mallet; and the ayes and nays being called for, the president said: it is a vote; when every man in the assembly said—"No vote, no vote, no vote." He then requested all who wanted the mallet removed into the center of the hall to stand up until counted. The excitement about moving the president, his mallet, and the other officers, with the entire stage into the center of the hall, had, by this time, become intense. While the counting was going on, a great rush from the outside was made, to learn, if possible, the cause of the disturbance, and the president continued to shout, with himself and mallet, that "All who were standing would be counted;" so that by the time the members in favor of not moving the stage, were asked to stand up for count, the hall was completely filled with eager inquirers for the disturbance, and were all counted, many on both sides, but enough on one side so that the president announced that the stage would not be moved. Five or six very loud and resolute men, called out at the very top of their voices: "Can't hear a word, can't hear a word; don't know anything what is said on the stage." The president modestly suggested, "That if one would halloo, at a time, their wind would probably go further." This completed the proceedings of the second day. The third and fourth days' sessions were more remarkable for results, and the hall was early crowded with members, mostly from the District of Columbia. Majority report only admitted to seats in the convention, a portion of the medical population of the District; and the minority report admitted the whole entire medical population of the District of Columbia. When it was moved to adopt the majority report, it was also moved to adopt the minority report; and about this time a sharp, resolute, earnest, member from Kentucky, moved that the District of Columbia be blotted from the map of the United States, seconded by half dozen associates. Pending the passage of this resolution, a distinguished member from Buffalo, N. Y., spoke earnestly about the dignity and honor of the great American Medical Association, and said that blotting out the District of Columbia was contrary to its objects and purposes, and that taking such action could only result in disaster and disgrace. He moved the resolution be laid on the table, which was carried by a unanimous vote. Colleges graduating women were, by some sort of resolution, introduced to the attention of the association, but as all *gentlemen* respect the rights and privileges of women, there was no division of the house. In the regular order of business, also, came colleges graduating negroes; and, pending this question, various motions and resolutions were introduced. This was late in the progress of affairs. and my recollections are rather indistinct. By this time, Washington and the neighboring districts were fully represented, and the question pending being the eligibility of colored men to vote, or rather the passage of the fifteenth amendment to the constitution, the highest point of mortal excitement was soon reached. The president, with his mallet, tried to preserve order. The secretary attempted to call the roll of members. Speeches upon the question were going on in all parts of the hall. Men were shouting at the top of their voices—" rise to order," " question of privilege;" Mr. Speaker, Mr. President, Mr. President; yes, yes; no, no, no. The secretary having read a list of names, the owners of which were on their way to, or anxiously looking for, their former homes, the president announced that the fifteenth amendment had *not passed;* while nearly the united voice of not only the convention, but of the entire country, and of history, said it *had* passed, and that " race or color shall not exclude" from, the right to vote. It may be said, in closing our account of the proceedings, that, all in all, it was one of the most harmonious and pleasant gatherings we have ever witnessed, and that the kindly recollections it has inspired will go with us through life.

